# Combination treatment with ABT-737 and chloroquine in preclinical models of small cell lung cancer

**DOI:** 10.1186/1476-4598-12-16

**Published:** 2013-03-02

**Authors:** Rebekah L Zinn, Eric E Gardner, Irina Dobromilskaya, Sara Murphy, Luigi Marchionni, Christine L Hann, Charles M Rudin

**Affiliations:** 1 Department of Oncology, The Sidney Kimmel Comprehensive Cancer Center at the Johns Hopkins University School of Medicine, Cancer Research Building 2, Room 544 1550 Orleans Street, Baltimore, MD, USA

**Keywords:** Autophagy, Apoptosis, ABT-737, Chloroquine, Primary xenograft

## Abstract

**Background:**

New therapies are urgently needed for patients with small cell lung cancer (SCLC). Chemotherapy and targeted therapies, including the Bcl-2 inhibitor ABT-737, may induce tumor cell autophagy. Autophagy can promote survival of cancer cells under stress and comprise a pathway of escape from cytotoxic therapies.

**Methods:**

We explored the combination of ABT-737 and chloroquine, an inhibitor of autophagy, in preclinical models of SCLC. These included cell culture analyses of viability and of autophagic and apoptotic pathway induction, as well as *in vivo* analyses of efficacy in multiple xenograft models.

**Results:**

Combination treatment of SCLC lines with ABT-737 and chloroquine decreased viability and increased caspase-3 activation over treatment with either single agent. ABT-737 induced several hallmarks of autophagy. However, knockdown of beclin-1, a key regulator of entry into autophagy, diminished the efficacy of ABT-737, suggesting either that the effects of chloroquine were nonspecific or that induction but not completion of autophagy is necessary for the combined effect of ABT-737 and chloroquine. ABT-737 and chloroquine in SCLC cell lines downregulated Mcl-1 and upregulated NOXA, both of which may promote apoptosis. Treatment of tumor-bearing mice demonstrated that chloroquine could enhance ABT-737-mediated tumor growth inhibition against NCI-H209 xenografts, but did not alter ABT-737 response in three primary patient-derived xenograft models.

**Conclusion:**

These data suggest that although ABT-737 can induce autophagy in SCLC, autophagic inhibition by choroquine does not markedly alter *in vivo* response to ABT-737 in relevant preclinical models, arguing against this as a treatment strategy for SCLC.

## Background

Autophagy is an evolutionarily conserved, reversible process in which the cell degrades its own cytoplasmic components utilizing lysosomal pathways. Typically, autophagy is activated under conditions of cell stress, including nutrient deprivation, environmental stressors, accumulation of misfolded proteins, and growth factor signal interruption. Cytoplasmic components are sequestered into double-membraned vesicles, or autophagosomes, which then fuse with lysosomes and the contained material is degraded to amino acids, nucleotides, sugars, and fatty acids that are released into the cytoplasm for biosynthesis and metabolism [1,2].

Recent evidence suggests that autophagy is frequently activated in tumor cells treated with chemotherapy and radiation [3-7]. While persistent activation of autophagy may lead to tumor cell death, accumulating evidence suggests that it can serve as a protective pathway for cells to escape apoptotic cell death, allowing survival through periods of stress, growth factor withdrawal, or nutrient deprivation [8,9]. Several studies have shown that autophagy delayed apoptotic death in cancer cells undergoing chemotherapeutic treatment. Treatment of these cells with inhibitors of autophagy, such as chloroquine, or knockdown of essential autophagy genes (beclin-1, ATG genes) resulted in enhanced therapy-induced apoptosis [10-14]. Chloroquine acts as a lysosomal pH inhibitor, therefore preventing the fusion of autophagosomes with lysosomes and inhibiting late stages of autophagy [15,16]. These findings have led to the initiation of multiple clinical trials combining autophagy inhibitors and chemotherapeutic agents for diverse cancer types [17].

Small cell lung cancer (SCLC) comprises approximately 15% of all lung cancers and is a remarkably aggressive disease [18]. SCLC is characterized by rapid proliferation and early dissemination to metastatic sites. While standard of care platinum-based chemotherapy induces responses in up to 80% of newly diagnosed SCLC cases, these responses are short-lived: the median survival in patients with advanced SCLC is approximately 9 months from the time of diagnosis [19]. New therapies are critically needed for this disease.

The anti-apoptotic protein Bcl-2 is overexpressed in the majority of SCLC cases [20,21]. Suppression of Bcl-2 can trigger apoptosis in SCLC, and enhance the anti-cancer efficacy of cytotoxic therapies [22]. ABT-737, a Bcl-2 homology domain 3 mimetic (BH3), which inhibits the activity of Bcl-2 and Bcl-X_L_, has been shown to have potent activity against many SCLC cell lines [23]. However, the efficacy of ABT-737 is variable across SCLC cell lines, and this agent has only modest single agent activity against most primary xenograft models of SCLC [24]. Consistent with the observed level of activity in primary xenografts, while clinical trials of ABT-263 (an orally bioavailable derivative of ABT-737 for human use) have shown pharmacodynamic evidence of on-target activity, the overall response rate in SCLC patients has been disappointing [25,26].

We hypothesized that SCLC resistance to ABT-737 may be in part due to cancer cell induction of autophagy. Bcl-2 has been shown to directly bind and inhibit Beclin-1 (*BECN1*), a critical mediator of initiation of autophagy [27]. Treatment of HeLa and MCF7 cells with ABT-737 or gossypol, another purported BH3 mimetic, has been shown to inhibit the interaction of Beclin-1 and Bcl-2/Bcl-X_L_ and stimulate autophagy [28-30]. We sought to define whether the treatment of SCLC cell lines with ABT-737 induces autophagy and whether this induction is a mechanism of therapeutic resistance. We also assessed the *in vivo* efficacy of the combination of ABT-737 and chloroquine using patient-derived xenograft (PDX) models of SCLC.

## Results

**Figure 1:**
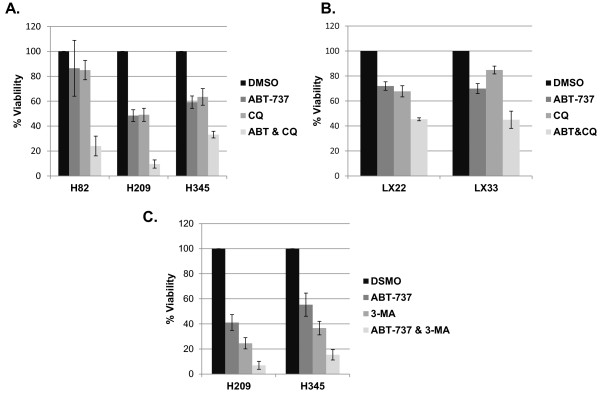
**Combination treatment of ABT-737 with autophagy inhibitors results in decreased proliferation in SCLC cell lines. **Cells were plated in 96-well plates and treated with DMSO, ABT-737, chloroquine (CQ), and the combination of ABT-737 and chloroquine. At 72 hours post-treatment they were assayed using the MTS cell proliferation assay. Commercially available cell lines are shown in **A **and cell lines derived from low passage cultures of primary patient xenografts in **B**. Doses chosen were based on initial determination of IC_50 _values and were as follows: H82 (17 μM ABT-737, 16.5 μM chloroquine), H209 (0.5 μM ABT-737, 20 μM chloroquine), H345 (0.25 μM ABT-737, 94 μM chloroquine), LX22 (6 μM ABT-737, 40 μM chloroquine), and LX33 (0.8 μM ABT-737, 33 μM chloroquine). **C**. Similar treatments were performed combining ABT-737 and 3-methyladenine, another autophagy inhibitor. 3-methyladenine doses were 5 mM for both lines. Error bars: standard deviation (SD); n = 4.

To assess whether an inhibitor of autophagy could affect viability of SCLC cell lines, and could augment the efficacy of the Bcl-2 inhibitor ABT-737, we initially treated several SCLC cell lines (H82, H209, and H345) with ABT-737, chloroquine, or the combination of both agents. Quantitative assessment of viable cells was performed at 72 hours using a standard MTS assay. In all three cell lines, the combination of ABT-737 with chloroquine significantly decreased cell viability relative to either agent alone (p < 0.002 for all three, see Figure 1A).

We have previously reported on a series of PDX tumors, generated by direct transfer of human tumor into immunosuppressed mice, which we have used as a preclinical platform for testing novel therapeutic strategies, including ABT-737 [24,31]. For correlative *in vitro* analyses, we have also generated derivative cell lines from two of these primary xenografts, LX22 and LX33. Cell lines derived from LX22 and LX33 both demonstrated greater loss of viability, as determined by MTS, in response to the combination of ABT-737 and chloroquine than to either agent alone (p < 0.003 for both, see Figure 1B).

These results suggested that inhibition of autophagy could enhance the efficacy of ABT-737. Alternatively, chloroquine could be having a cytotoxic effect unrelated to inhibition of autophagy in these cells. We interrogated this intersection between apoptotic and autophagic pathways using an alternative pharmacologic inhibitor of autophagy, 3-methyladenine (3MA). Treatment with 3MA, with or without ABT-737, in H209 and H345 cells resulted in similar results to those obtained with chloroquine: clear evidence of enhanced cytotoxic response in cells treated with the combination relative to either agent alone (p < 0.0015 for both, see Figure 1C).

**Figure 2:**
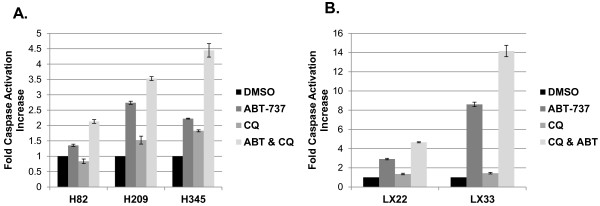
**Combination of ABT-737 and chloroquine results in increased induction of apoptosis in SCLC cell lines. **Cells were treated at the doses listed in Figure 1 for ABT-737 and chloroquine or the combination for 72 hrs, and then assayed using the Caspase-Glo 3/7 assay. Luminescence is directly proportional to the amount of caspase-3 activation. Commercially available cell lines are shown in **A** and cell lines derived from patient xenografts are shown in **B**. Error bars: SD; n = 4.

We next sought to characterize whether the combinatorial effects observed in cells treated with ABT-737 and chloroquine were associated with enhanced apoptosis, as hypothesized. Caspase-3 activation is a central hallmark of apoptotic induction through both the extrinsic and intrinsic apoptotic pathways. The combination of ABT-737 and chloroquine resulted in higher levels of caspase-3 activation relative to either treatment alone in several SCLC lines (Figure 2A), and in the cell lines derived from primary patient xenografts (Figure 2B). Chloroquine consistently resulted in less caspase-3 activation than ABT-737, and in some lines – including both lines derived from primary patient xenografts – led to almost no caspase-3 activation as a single agent, yet substantially increased activated caspase-3 when combined with ABT-737.

**Figure 3:**
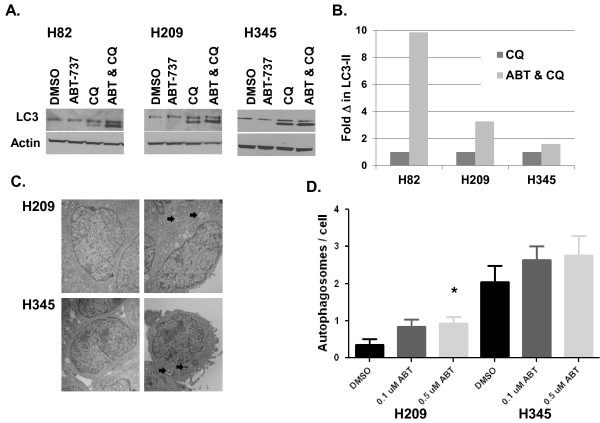
**ABT-737 Treatment induces autophagy in SCLC cell lines. A**. Cell lines were treated with doses of ABT-737, CQ, or the combination at doses indicated in Figure 1 for 72 hrs. Protein lysates were harvested and western blots performed using antibodies for LC3 and β-actin. **B. **LC3-II band intensities from the Western blots shown in A of cells treated with CQ vs. ABT & CQ, normalized to Actin as a loading control. **C**. H209 and H345 cells were vehicle treated with DMSO (left panels) or treated with ABT-737 (H209, 0.1 μM ABT-737, H345 0.5 μM ABT-737, right panels) for 72 hrs and harvested for electron microscopy. Block arrows indicate autophagosomes. **D. **The number of autophagosomes per cell prolife was counted for 25 randomly chosen profiles from each sample and averaged. * p < 0.05 relative to DMSO vehicle.

Upon activation of autophagy, the LC3-I protein is phosphatidylethanolamine (PE) conjugated to form LC3-II and is preferentially translocated to the membranes of autophagosomes. The appearance of an LC3-II band on western blots is a generally accepted indicator of autophagy [32]. If apoptotic induction by ABT-737 leads to a protective autophagic response in cancer cells, then we might expect to see detectable markers of upregulated autophagy following ABT-737 exposure, particularly in the context of decreased flux through the autophagic pathway by concomitant treatment with chloroquine. To test this idea, we performed similar single agent and combination drug treatments, and isolated protein to blot for levels of LC3-II. No LC3-II was detectable in vehicle control-treated cells, or in cells treated with single agent ABT-737, but was clearly evident in all cell lines when treated with chloroquine, and further enhanced by the combination of chloroquine and ABT-737 (Figure 3A). Measurement of LC3-II levels showed that for each cell line, there was an increase in LC3-II in the combination treatment compared to chloroquine alone (Figure 3B). These data suggest that ABT-737 may in fact trigger autophagy, as previously suggested [33], but that flux through the pathway precludes detection of LC3-II unless an inhibitor of completion of autophagic digestion such as chloroquine is present. To more conclusively assess whether ABT-737 induces autophagy, we examined ABT-737 treated cell lines by transmission electron microscopy (TEM) to look for the presence of characteristic autophagosomes. Representative images shown in Figure 3C and quantitative data presented in Figure 3D confirm a small dose-dependent increase in autophagosome formation in response to ABT-737 in both H209 and H345 cells (Figure 3C and D). However, statistical significance was only reached in H209 when comparing 0.5 μM ABT-737 treatment to control (p = 0.025).

**Figure 4:**
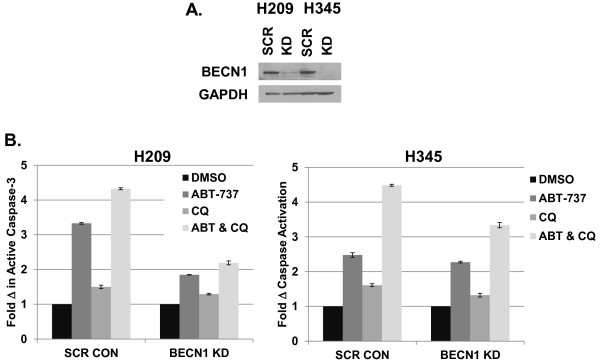
**Knockdown of Beclin-1 diminishes the effect of ABT-737. A**. shRNA knockdown of *BECN1 *(KD) or scrambled control (SCR) was performed in H209 and H345 cells. After puromycin selection, cell lysates were harvested and Western blot analysis was performed using antibodies specific for Beclin-1 or GAPDH.** B**. Cells were treated with ABT-737 (0.1 μM), CQ (12.5 μM) or the combination for 72 hrs and then assayed using the Caspase-Glo 3/7 kit. The y-axis shows fold differences in caspase activation between treatments compared to DMSO treated samples. Error bars: SD; n = 4.

While both chloroquine and 3MA appeared to enhance the efficacy of ABT-737 against SCLC, these pharmacologic agents could both be influencing cell proliferation and apoptosis via pathways other than autophagy. In addition, chloroquine may affect completion of autophagy in cells committed to autophagic induction, rather than influencing initiation of this pathway. To further dissect the potential interactions between the apoptosis and autophagy pathways in SCLC, we adopted a genetic approach, using shRNA knockdown of *BECN1* in both H209 and H345 cells (Figure 4A). Beclin-1 is of particular interest, both an essential autophagy protein that controls the first steps of autophagic commitment, and as a known binding partner of the primary targets of ABT-737, Bcl-2 and Bcl-x_L_. Surprisingly, and in sharp contrast to the results obtained with chloroquine and 3MA, knockdown of BECN1 decreased caspase-3 activation in response to ABT-737 and the combination of ABT-737 and chloroquine (Figure 4B).

Similar suppressive effects on apoptotic induction were seen in H209 and H345. Taken together with data demonstrating that ABT-737 can activate autophagy (as determined by LC3-II levels and autophagosome formation), and that two inhibitors of autophagy (chloroquine and 3MA) can augment ABT-737 efficacy, the entirely unexpected results obtained using *BECN1* knockdown suggest that the complex of Bcl-2 and Beclin-1 may have functional significance in the regulation of apoptosis by Bcl-2, independent of the suppressive effect on autophagy.

**Figure 5:**
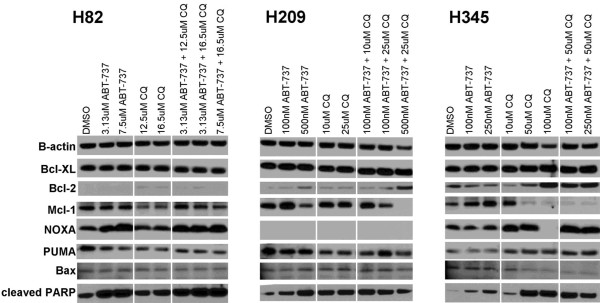
**ABT-737 and chloroquine combination therapy alters the expression levels of apoptotic regulatory factors in SCLC. **The cell lines H82, H209 and H345 were treated with DMSO (control), ABT-737, CQ, and combinations of the two drugs for 72 hours before protein lysates were isolated. Doses of ABT-737 and CQ that were below or approximating the cell line specific IC_50 _values for each agent were chosen. Beta-actin is shown as an internal loading control and cleaved-PARP is shown for relative induction of apoptosis. NOXA was undetectable in H209 and Bcl-2 was barely detectable in H82.

It is possible that the inhibitors of autophagy used here could have biological effects on the regulation of cell survival independent of the autophagy pathway. Notably, resistance to ABT-737 in SCLC cell lines has been associated with increased expression of the anti-apoptotic Bcl-2 family member Mcl-1, which is not effectively inhibited by ABT-737, and decreased expression of NOXA, a pro-apoptotic Bcl-2 family member which inhibits Mcl-1 [34]. What is more, both chloroquine and 3-MA have been shown to decrease Mcl-1 expression in neutrophils [35]. Together, these observations suggest an alternative and potentially complementary mechanism by which the combination of ABT-737 and chloroquine could effectively inhibit SCLC growth. To explore whether these mechanisms might be operant in our cell lines, we performed Western blotting of Mcl-1, NOXA, and other relevant Bcl-2 family members in H82, H209, and H345 cells treated with ABT-737, chloroquine, or combinations of both agents. As seen in Figure 5, in at least two of these lines (H209 and H345), combination therapy was associated with marked decrease in Mcl-1. H209 did not express detectable NOXA under any conditions, but in the other two lines, combination therapy was also associated with NOXA upregulation. These data suggest that at least part of the combinatorial effect of ABT-737 and chloroquine may be mediated by alteration in the relative balance of pro- and anti-apoptotic Bcl-2 family members, promoting cell death.

**Figure 6:**
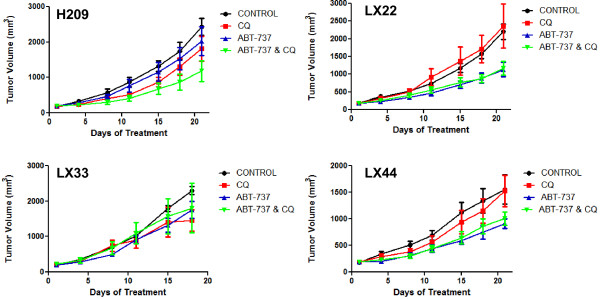
**Combination of ABT-737 and chloroquine does not result in increased efficacy in xenografts models. **Tumor bearing Nu/Nu mice (4–7 per treatment arm) were treated daily with ABT-737 (100 mg/kg, IP),CQ (60 mg/kg, IP), combination, or an equal amount of vehicle control for 18–21 days. Tumor volumes were measured every three days. Group means and standard error of the mean for each measurement are shown.

Finally, given the observed enhanced cytotoxicity seen *in vitro* through combination of ABT-737 and chloroquine, we sought to explore whether this combination could improve tumor control *in vivo*. Cohorts of established tumor xenografts of the cell line H209, as well as three SCLC PDX models, LX22, LX33, and LX44, were treated with daily intraperitoneal injection of chloroquine, ABT-737, combination, or vehicle-treated controls, and tumor volume was monitored over time. The combination was superior to single agent treatment in the H209 xenografts (p = 0.005 for comparison with ABT-737 alone), but showed no evidence of superiority over ABT-737 alone in the treatment of the primary xenograft models (p > 0.5 for comparison with ABT-737 alone in the primary xenografts) (Figure 6). The interaction p-value remains significant for H209 with a Bonferroni correction for multiple testing (p = 0.02).

## Discussion

In this study we sought to assess whether the efficacy of the targeted Bcl-2 inhibitor, ABT-737, against SCLC could be improved by concurrent inhibition of autophagy with the clinically available agent chloroquine. *In vitro* analyses of the combination of ABT-737 and chloroquine in several SCLC cell lines did show a decrease in viability and a concomitant increase in caspase-3 activation. In addition, we found evidence that ABT-737 induced autophagy in several cell lines by the conversion of LC3-I to LC3-II seen on western blots and increased autophagosome formation as documented by TEM.

However, a targeted genetic approach to blocking entry into autophagy based on shRNA knockdown of *BECN1* yielded the opposite result: a consistent decrease in apoptotic response to ABT-737, with or without chloroquine. There are several possible explanations for these seemingly contradictory results. First, the effects seen with chloroquine may not have been specific to the autophagy pathway. Indeed, we were able to demonstrate that the combination of ABT-737 and chloroquine can lead to marked inhibition of Mcl-1 and upregulation of NOXA, both of which would serve to facilitate apoptotic induction, independent of an effect on autophagy. It is known that chloroquine can also act on several other pathways including those involved in immune modulation and DNA damage [17].

Second, while chloroquine inhibits the late stages of autophagy, i.e. the fusion of autophagosomes with lysosomes leading to degradation of vesicular contents, Beclin-1 is required for an initial commitment toward autophagy. It is possible that successful initiation of autophagy without the ability to complete the process leads to intracellular accumulation of toxic materials, leading to enhanced apoptotic cell death, while blockade of entry into this pathway does not. Some reports do suggest that specific stage of autophagy inhibition can result in different therapeutic outcomes [5,36]. Our data with 3MA would argue against this explanation to some extent: 3MA inhibits initiation of autophagy at a stage closely related to that of Beclin-1, but unlike Beclin-1 suppression, augmented the apoptotic response to ABT-737 in multiple lines. 3MA is a class I and class III PI3K inhibitor [14], and could, like chloroquine, have other relevant effects leading to enhanced programmed cell death.

Third, and perhaps most intriguingly, Beclin-1 and Bcl-2 are known to directly interact, and shRNA suppression of *BECN1* may affect Bcl-2 function or localization independent of any effect on induction of autophagy. While previous data suggest that Beclin-1 does not affect the anti-apoptotic activity or localization of Bcl-2, this finding may be context and cell type-specific; these studies were performed in HeLa cervical carcinoma cells and mouse embryonic fibroblasts [37]. One possible model to explain our results would be if Beclin-1 sequesters some fraction of the cellular Bcl-2 away from the mitochondrial membrane, where Bcl-2 primarily acts to suppress outer mitochondrial membrane disruption. Targeted loss of Beclin-1 could lead to a preferential localization of Bcl-2 to mitochondria, decreasing ABT-737 efficacy by functional upregulation of the target. The novel observations in this study suggest several testable hypotheses to be addressed in future studies, which may provide additional insights into the interface between regulators of apoptosis and autophagy in SCLC.

## Conclusions

Several ongoing clinical trials seek to evaluate the efficacy of autophagy inhibitors in combination with cytotoxic therapies in cancer types including SCLC. The data presented here have important implications for studies in SCLC. Different strategies for modulating autophagy can have markedly discordant effects on the efficacy of cytotoxic therapies. It will be important to study the modulation of autophagy closely in relevant preclinical models to anticipate possible combinatorial effects.

In work presented here, we analyzed for the first time the effect of combining ABT-737 and chloroquine in SCLC models, including assessment of activity against PDX tumors, models that may better reflect the biology of disease than do cell line-based xenografts [31,38]. Our *in vivo* xenograft data, particularly in the three primary xenograft models, suggest that despite some intriguing molecular interactions, the combination of ABT-737 and chloroquine is unlikely to have significant anti-cancer activity against SCLC, relative to ABT-737 alone.

## Methods

### Cell lines

All cell lines were obtained from American Type Culture Collection and grown in the recommended media. Primary xenograft cell lines (LX) were derived from patient tumors as previously described [31].

### MTS cell proliferation assays

Cells were plated in quadruplicate and treated with ABT-737 and chloroquine for 72 hours at indicated doses. MTS cell proliferation assays were performed following manufacturer instructions using the CellTiter 96 AQ_ueous_ One Solution Cell Proliferation Assay (Promega).

Caspase-Glo 3/7 assay. Assay was performed following manufacturer instructions (Promega). Briefly, 20,000 viable cells from each sample were transferred to 96-well white-walled plates in 75–100 μl total volume of culture medium. An equal volume of Caspase-Glo 3/7 Reagent was added to each well. Assays were performed in quadruplicate. Plates were covered and incubated at room temperature for 1–2 hours before measuring luminescence on a plate reading luminometer. The luminescence reading is directly proportional to caspase-3 activity.

### Western blotting

Protein was isolated from cell pellets using radioimmunoprecipitation assay buffer (Sigma) and run on 10 or 12% Bis-Tris gels with MOPS or MES running buffer (Invitrogen). Membranes were probed with antibodies for LC3, Beclin-1, GAPDH, β-Actin (Santa Cruz), Mcl-1 (BD Pharmigen; #559027), Bax (Santa Cruz; #sc-6236), Bcl-XL (Cell Signaling; #2764), Bcl-2 (Santa Cruz; #sc-492), NOXA (Imgenex; #IMG-349A), PUMA (Cell Signaling; #4976), and cleaved PARP (Cell Signaling; #5625) following manufacturer instructions.

### Electron microscopy

Samples were fixed in 2.5% glutaraldehyde, 3 mM CaCl_2_, 1% sucrose, in 0.1 M sodium cacodylate buffer, pH 7.2 for one hour at room temperature. After buffer rinse, samples were postfixed in 1% osmium tetroxide in buffer (1 hr) on ice in the dark. Following a DH_2_O rinse, plates were stained with 2% aqueous uranyl acetate (0.22 μm filtered, 1 hr, dark), dehydrated in a graded series of ethanol and embedded in Eponate 12 (Ted Pella) resin. Samples were polymerized at 60°C overnight. Thin sections, 60 to 90 nm, were cut with a diamond knife on the Reichert-Jung Ultracut E ultramicrotome and picked up with naked 200 mesh copper grids. Grids were stained with 2% uranyl acetate in 50% methanol and observed with a Philips CM 120 at 80 kV. Images were captured with an Advanced Microscopy Techniques transmission electron microscopy (1 K × 1 K) camera.

### Lentiviral shRNA and cDNA overexpression experiments

Lentiviral particles were generated using a three-plasmid system and infected as per the RNAi Consortium Library Production and Performance Protocols, Broad Institute [39]. shRNA constructs were obtained from the Broad RNAi Consortium and screened for knockdown. The *BECN1* shRNA clone ID TRCN0000033550 was used these experiments. pLKO.1-shRNA scramble vector was obtained from Dr. David M. Sabatini through Addgene (Addgene plasmid 1864) as previously described [40].

### *In vivo* studies

Patient-derived xenograft tumor models LX22, LX33, and LX44 were generated as previously described [24,31]. NCI-H209 cells (1 × 10^7^), or SCLC patient derived tumor cells LX22 (1.5 × 10^6^), LX33 (1.6 × 10^6^), and LX44 (3.5 × 10^6^), were suspended in a 1:1 ratio of PBS and matrigel (BD Biosciences). Cell suspensions were injected subcutaneously on right hind flanks of female nu/nu 6 – 8 week old mice (Charles River). Drug treatments began when tumor sizes reached 200 mm^3^. Mice were treated daily with 100 mg/kg ABT-737 (IP), 60 mg/kg chloroquine (IP), combination or vehicle treated for 21 days. Tumors were measured with a manual caliper every 3 days and volumes were calculated using the formula: tumor weight (mg) = [length (mm) x width^2^ (mm^2^)]/2. In order to test whether treatment inhibited tumor growth, we used a mixed effects linear model comparing tumor growth between ABT-737 and ABT-737 and CQ treatment groups, modeling the log of tumor size over time and including an interaction term. All *in vivo* experiments were conducted with protocol approval by the Johns Hopkins Animal Care and Use Committee.

### Statistical analyses

The student’s *t*-test was used to compare combination treatment effects vs. single agent treatment effects in cell lines. For *in vivo* analyses of whether treatment inhibited tumor growth, we used a mixed effects linear model comparing tumor growth between treatment groups, modeling the log of tumor size over time and including an interaction term. A p value <0.05 was considered significant.

## Abbreviations

3MA: 3-Methyladenine; CQ: Chloroquine; MTS: (3-(4,5-dimethylthiazol-2-yl)-5-(3-carboxymethoxyphenyl)-2-(4-sulfophenyl)-2H-tetrazolium); PDX: Patient-derived xenograft; SCLC: Small cell lung cancer; TEM: Transmission electron microscopy

## Competing interests

The authors have no competing interests with regard to the data presented here.

## Authors’ contributions

RLZ conducted the majority of experiments reported here, and drafted the manuscript. EEG conducted additional essential *in vitro* experiments. ID assisted with all *in vivo* experiments. SM assisted with molecular and cell culture studies. LM provided statistical support. CLH provided key intellectual input, practical advice on experimental approaches, and assisted with data interpretation. CMR conceived of the study, participated in its design and coordination, and assisted with data interpretation and manuscript writing. All authors read and approved the final manuscript.
